# Antimicrobial Resistance in *Escherichia coli* Isolated from Healthy Dogs and Cats in South Korea, 2020–2022

**DOI:** 10.3390/antibiotics13010027

**Published:** 2023-12-27

**Authors:** Bo-Youn Moon, Md. Sekendar Ali, Dong-Hyeon Kwon, Ye-Eun Heo, Yu-Jeong Hwang, Ji-In Kim, Yun Jin Lee, Soon-Seek Yoon, Dong-Chan Moon, Suk-Kyung Lim

**Affiliations:** 1 Bacterial Disease Division, Animal and Plant Quarantine Agency, 177 Hyeksin 8-ro, Gimcheon-si 39660, Republic of Korea; qiamby@korea.kr (B.-Y.M.); alipharm@iiuc.ac.bd (M.S.A.); kwondh7719@korea.kr (D.-H.K.); gdd0707@korea.kr (Y.-E.H.); dbwjd0386@korea.kr (Y.-J.H.); j11506@korea.kr (J.-I.K.); sky10bulu@korea.kr (Y.J.L.); yoonss24@korea.kr (S.-S.Y.); 2 Division of Antimicrobial Resistance Research, Centre for Infectious Diseases Research, Korea Disease Control and Prevention Agency, Cheongju 28159, Republic of Korea

**Keywords:** cephalosporins, quinolones, multidrug resistance, companion animals

## Abstract

The occurrence of antimicrobial-resistant bacteria in companion animals poses public health hazards globally. This study aimed to evaluate the antimicrobial resistance profiles and patterns of commensal *E. coli* strains obtained from fecal samples of healthy dogs and cats in South Korea between 2020 and 2022. In total, 843 *E. coli* isolates (dogs, *n* = 637, and cats, *n* = 206) were assessed for susceptibility to 20 antimicrobials. The resistance rates of the most tested antimicrobials were significantly higher in dog than in cat isolates. Cefalexin (68.9%) demonstrated the highest resistance rates, followed by ampicillin (38.3%), tetracycline (23.1%), and cefazolin (18.7%). However, no or very low resistance (0–0.6%) to amikacin, imipenem, piperacillin, and colistin was found in both dog and cat isolates. Overall, 42.3% of the isolates exhibited multidrug resistance (MDR). MDR in isolates from dogs (34.9%) was significantly higher than in those from cats (20.9%). The main components of the resistance patterns were cefalexin and ampicillin in both dog and cat isolates. Additionally, MDR patterns in isolates from dogs (29.2%) and cats (16%) were shown to encompass five or more antimicrobials. Multidrug-resistant commensal *E. coli* could potentially be spread to humans or other animals through clonal or zoonotic transmission. Therefore, the incidence of antimicrobial resistance in companion animals highlights the urgent need to restrict antimicrobial resistance and ensure the prudent use of antimicrobials in Korea.

## 1. Introduction

*Escherichia coli* is a facultative aerobic and anaerobic commensal Gram-negative bacterium that resides in the gastrointestinal tract of humans and other animals. Commensal *E. coli* are frequently exposed to antimicrobial agents during the lifespan of their host. Consequently, commensal strains acquire resistance genes and/or undergo mutations that provide resistance, enabling them to survive and maintain microbial homeostasis inside the digestive tract. Thus, commensal *E. coli* strains are considered to be indicators of the level of antimicrobial exposure in their hosts [1]. The issue of antimicrobial resistance (AMR) is a significant concern within the fields of human and veterinary medicine. Compared to their susceptible counterparts, resistant bacterial infections are linked to higher morbidity, mortality, and treatment costs [2]. The presence of antibiotic-resistant commensal *E. coli* strains, associated with pathogenicity, serves as a reservoir for AMR genes. Transfer of these genes has the potential to occur in humans and other animals, limiting the available treatment options [3].

Antimicrobial resistance in *E. coli* has been frequently demonstrated in companion animals worldwide. It was found that *E. coli* isolates obtained from companion animals in different countries, including Japan [4], Iran [5], Australia [6], Belgium [7], Portugal [8], the U.K. [9], Canada [10], and the U.S.A. [11], exhibited resistance to various antimicrobial agents, including those deemed to be critically important for humans. The misuse and/or overuse of antimicrobial agents in both humans and animals present a critical concern regarding the possible emergence of antibiotic resistance in the commensal bacteria *E. coli* [12]. The possibility of antimicrobial resistance in *E. coli* allows for their transmission from companion animals such as dogs and cats to humans, either directly or indirectly, as they share the same environment as humans, remain in close proximity, and are also exposed to antimicrobials for therapeutic purposes, which are commonly used for humans [13]. For example, Belas et al. [14] showed that clinically important AMR genes in *Enterobacteriaceae* were transmitted between healthy companion animals and humans.

It is possible to compare the prevalence of resistance in different populations and identify any potential transmission of resistant bacteria from healthy companion animals to humans and vice versa by looking into the prevalence of resistance in specific indicator bacteria, such as *E. coli* and enterococci, in the intestinal tracts of various populations of humans and healthy companion animals [14].

Several investigations were carried out in Korea to evaluate the level of antibiotic resistance in *E. coli* isolated from humans and companion animals [15,16,17]. However, very few data were obtained from healthy companion animals. As antimicrobial-resistant *E. coli* can be transmitted due to frequent interactions between humans and their healthy companion animals, we aimed to ascertain the antimicrobial sensitivity and resistance patterns of *E. coli* isolated from healthy companion animals such as dogs and cats in South Korea from 2020 to 2022.

## 2. Results

### 2.1. Antimicrobial Resistance Rate

The resistance rate of the isolated *E. coli* against 20 antimicrobials is presented in Table 1 and Table S2. The isolates’ highest resistance rates were observed for cephalexin (68.9%), followed by ampicillin (38.3%), tetracycline (23.1%), and cefazolin (18.7%). However, the remaining antimicrobials, i.e., amikacin, amoxicillin, ceftazidime, gentamicin, imipenem, piperacillin, and colistin, showed low resistance rates (<15%) and very low resistance rates (0.1–7.4%). With respect to the animal species, the overall prevalence of antibiotic resistance was significantly higher in *E. coli* obtained from dogs than in samples from cats (*p* < 0.05). Among the tested antimicrobials, most isolates (>50%) were resistant to cefalexin, and cefalexin resistance rate was higher in dog isolates (74.4%) than in cat isolates (51.9%). Moreover, the dog isolates were found to have higher rates of resistance to ampicillin (42.4 vs. 25.7%), tetracycline (25.6 vs. 15.5%), and cefazoline (20.4 vs. 13.6%) than those from cats. However, no or meager (less than 1%) resistance to amikacin, imipenem, piperacillin, and colistin was observed in isolates from both dogs and cats. The MIC_50_ and MIC_90_ values of the investigated antimicrobials are presented in Table S1.

The *E. coli* isolates obtained from dogs and cats exhibited varying levels of antimicrobial resistance according to their hosts’ age (Table 2). Overall, compared to the isolates from puppies and juveniles (aged 1 year), mature adults (aged 2–5 years), and geriatric groups (aged ≥11 years), the isolates from senior dogs (aged 6–10 years) exhibited a slightly higher antimicrobial resistance rate. But the difference was not significant. Resistance also varied by antimicrobial. In dogs, resistance to ampicillin, doxycycline, tetracycline, and trimethoprim/sulfamethoxazole was high in the young age group (less than 5 years of age). Notably, resistance to critically important antimicrobials for humans was high in the older age group. Resistance to third-generation cephalosporins (cefovecin) and fluoroquinolones (enrofloxacin, marbofloxacin, and orbifloxacin) was high in the isolates from the senior and geriatric groups. In cats, generally, the isolates from the mature group (aged 7–10 years) showed a higher antimicrobial resistance rate than the isolates obtained from juniors and adults (aged 2–6 years) and from kittens (aged ≤1 year). Especially, resistance to β-lactam antimicrobials (ampicillin, amoxicillin/clavulanic acid) and third-generation cephalosporins was higher than that to other antimicrobials. Of note, imipenem resistance was detected in isolates from dogs but not in those from cats.

### 2.2. Multidrug Resistance (MDR) and Antimicrobial Resistance Patterns

In this investigation, it was found that 85.1% (542/637) of the dog isolates and 57.8% (119/206) of the cat isolates displayed resistance to at least one antimicrobial (Table 3 and Table 4). In total, 42.3% of the isolates exhibited multidrug resistance (MDR). MDR was significantly higher in the isolates obtained from dogs (34.9%) as compared to the isolates from cats (20.9%) (*p* < 0.05). Resistance to five or more antimicrobials was found in 29.2% of dog isolates and 16% of cat isolates.

The isolates from dogs and cats were found to possess a total of 152 and 42 MDR combination patterns, respectively (Tables S3 and S4). Moreover, the main components of the patterns were β-lactam antimicrobials. Among them, resistance to cefalexin was the type of resistance most frequently recorded in both dogs (31.9%) and cats (28.2%) (Table 3 and Table 4).

## 3. Discussion

In this investigation, it was observed that a major proportion of *E. coli* isolates exhibited resistance to cefalexin. Consistent with previous reports [18,19], we found high cefalexin resistance rates in *E. coli* recovered from dogs and cats. Furthermore, significant resistance rates were also noted for ampicillin, tetracycline, and cefazolin in *E. coli* isolated from different countries, including Korea [15] and the U.S.A. [11]. The extensive use of these antimicrobial agents in companion animals exerts selective pressure, triggering the development of resistance in *E. coli*. 

Cephalosporins are among the important antimicrobials used for the treatment of multidrug-resistant bacterial infections in humans [20]. The present investigation observed that the rates of cephalosporin resistance among the isolates from dogs and cats varied, ranging from 5.7 to 68.9%. Among them, high cefalexin resistance rates (>50%) were found in *E. coli* isolates recovered from dogs (74.4%) and cats (51.9%). The high cefalexin resistance rates in companion animals are consistent with earlier findings in Spain (66.1%) [21], Italy (100%) [22], and the U.S.A. (98%) [23]. In contrast, a relatively low prevalence of cefalexin resistance in *E. coli* isolates obtained from dogs in Brazil (33%) was reported [24]. Moreover, we found a moderate cefazolin resistance rate (18.7%) in dog and cat isolates, comparatively lower than that reported by Harada et al. [25] in Japan (31.7%). However, the rates of cefazolin resistance observed in the examined dog and cat isolates were found to be similar to those in previous reports from Korea (18%) [26]. The reason for this resistance to cefalexin might be the heavy use of this antimicrobial. Cefovecin is one of the most commonly used third-generation cephalosporins in companion animals in Korea. In the current investigation, it was observed that 16.2% and 9.7% of the *E. coli* strains obtained from dogs and cats exhibited resistance to cefovecin. The rate of cefovecin resistance seen in this investigation aligns with that reported in a previous study conducted in the U.K. (20%) [27]. It was, though, lower than that measured in previous reports from Chile (30.4%) [28] and Canada (94.4%) [29]. Similarly, the cefpodoxime resistance rates found in the dogs (16.5%) and cat (9.7%) isolates are consistent with those determined in studies conducted in Argentina (16%) [30] and Nigeria (9.7%) [31]. The prevalence of cephalosporin resistance may be related to the frequent use of these antimicrobials to treat infections in companion animals [32]. This may decrease the possibility to use of these antimicrobial agents, which are essential for the treatment of severe infections.

Quinolones are among the crucial antimicrobial agents used for the treatment of infections in humans and animals [33]. However, the elevated occurrence of fluoroquinolone-resistant *E. coli* in both companion animals and humans is worrying. In accordance with a previous investigation [34], we observed a higher prevalence of resistance to enrofloxacin and marbofloxacin in dog isolates (16.2% each) than in cat isolates (6.3% each). Similar to this, prior investigations from Korea [35] and the U.K. [36] revealed high incidences of enrofloxacin and/or marbofloxacin resistance more frequently in dog isolates than in cat isolates. In particular, the enrofloxacin resistance rate in this study was higher than that reported in Canada (4.5% and 2.4% in isolates from dogs and cats, respectively) [29]. We also observed higher resistance rates for orbifloxacin and pradofloxacin in dog isolates (17.4% and 15.7%, respectively), which were more than two times greater compared to those found in cat isolates (7.3% and 6.3%, respectively). The observation of these antimicrobials (orbifloxacin and pradofloxacin) resistance rates in dog isolates concurs with an earlier study conducted in the U.S.A. (21.3 and 23.3%, respectively) [37]. Nevertheless, the prevalence of orbifloxacin and pradofloxacin resistance in dog (89 and 90%, respectively) and cat (97% each) isolates reported by KuKanich et al. [38] was higher than in our study. The increased resistance to these antimicrobials in *E. coli* strains from companion animals is a matter of concern. Moreover, the cross-resistance of fluoroquinolones can affect public health, as fluoroquinolones in companion animals foster the development of bacterial resistance that can be transmitted to humans. For example, the potential transfer of ciprofloxacin-resistant *E. coli* from companion animals to humans has been hypothesized, even though ciprofloxacin is not used in animals, and can be explained by the occurrence of cross-resistance between fluoroquinolones, as well as by the fact that enrofloxacin, a frequently used antimicrobial in companion animals, undergoes partial metabolism to ciprofloxacin in animals [39]. 

Carbapenems are the last-resort antimicrobial agents for treating infections caused by multidrug-resistant bacteria [40]. However, the global emergence of carbapenemase-producing bacteria is an alarming indication, potentially leading to ever-increasingly restricted therapeutic choices. In our study, we identified imipenem-resistant *E. coli* in dog isolates (0.1%). In Korea, imipenem-resistant *E. coli* carrying the *bla*_NDM-5_ gene were reported in dogs and cats [41]. Moreover, imipenem-resistant *E. coli* isolates were detected in dogs in the U.S.A. (0.6%) [42], Japan (1.6%) [4], Spain (<1%) [21], and China (25%) [43]. 

The prevalence of *E. coli* that have developed resistance to critically important antimicrobials such as colistin presents a global health hazard. In this study, colistin resistance was detected in dogs (0.1%). Previous studies from Asian [44] and European countries [45,46] revealed low proportions of isolates from dogs resistant to colistin (0.1–2%), which is consistent with our findings. In contrast, a comparatively high prevalence of resistance to colistin in dog isolates from Slovakia (5.3%) was documented [47]. The plasmid-mediated mobile colistin resistance gene *mcr-1*, contributing to the development of colistin resistance in *E. coli*, was identified in companion animal dogs in our previous study [48].

In order to understand the emergence of antimicrobial resistance and develop treatment plans and preventive measures for companion animals of various ages, the prevalence of antimicrobial-resistant *E. coli* among dogs and cats depending on age was assessed. In this investigation, we noted that resistance to important antimicrobials (third-generation cephalosporins and fluoroquinolones), mainly used as second or third antimicrobial agents, was found in dogs in older age groups. Similarly, for cats, the isolates from the mature group (aged 7–10 years) showed a greater antimicrobial resistance rate than those from other age groups. However, the isolates from puppies and juveniles (aged 1 year) showed higher resistance to cefalexin, ampicillin, doxycycline, tetracycline, and trimethoprim/sulfamethoxazole compared to those from mature adults, senior, and geriatric dogs. In agreement with prior research, this study revealed a higher incidence of antimicrobial resistance in isolates collected from companion animals of older age [49]. The likelihood of increased antimicrobial resistance in companion animals of advanced age may be attributed to their prolonged exposure to more therapeutic drugs throughout their lifespan [50]. However, earlier research on the antimicrobial resistance patterns in isolates obtained from animals of various ages showed inconsistent outcomes [51].

The majority of *E. coli* isolates demonstrated resistance to at least one antimicrobial agent, with multiple resistance patterns reported for isolates obtained from both dogs and cats. This study found higher multidrug resistance (MDR) rates in dog (34.9%) compared to cat (20.9%) isolates. A similar MDR rate was seen in *E. coli* isolated from dogs (14.5%) and cats (13.3%) in Canada [29]. Nevertheless, it was documented that the prevalence of MDR in *E. coli* isolates recovered from dogs and cats was higher in China (63.7%) [52] and the U.S.A. (56%) [53] than in our current study. These outcomes imply that high selective pressures for antibiotic resistance are present in companion animals. Additionally, the emergence of MDR in *Enterobacteriaceae* is further exacerbated by the existence of mobile genetic elements, including plasmids and transposons [54,55]. Hong et al. [15] showed that *E. coli* isolated from healthy dogs and cats carried a variety of plasmids harboring mobile genetic elements and genes for antibiotic resistance, demonstrating that the observed resistance may be caused by plasmid transfer.

## 4. Materials and Methods

### 4.1. Isolation and Identification of E. coli

The isolation and identification of *E. coli* from the feces of healthy (with no clinical signs and symptoms of disease) dogs and cats were conducted using the procedures described in our previously published study [56]. Briefly, the fecal samples were applied onto Eosin Methylene Blue (EMB) agar (Becton Dickinson, Sparks, NV, USA) and subjected to incubation at 37 °C for 24 h. The colonies were then sub-cultured on MacConkey agar plates (MAC, BD, Spark, Baltimore, MD, USA) by incubating them overnight at 37 °C. The isolates were subsequently confirmed using matrix-assisted laser desorption and ionization-time-of-flight mass spectrometry (MALDI-TOF, Biomerieux, Marcy L’Etoile, France). One isolate per animal was analyzed in this study. A total of 843 *E. coli* isolates (637 from dogs and 206 from cats) were obtained from 8 laboratories/centers participating in the Korean Veterinary Antimicrobial Resistance Monitoring System (KVARMS) between 2020 and 2022 (Table 5). The collected isolates contained in 30% glycerol were preserved at −70 °C until subsequent analysis. No ethical approval was deemed necessary for this study.

### 4.2. Antimicrobial Susceptibility Assessment

The antimicrobial susceptibility assessment was carried out by the broth microdilution method using the commercially available sensititre plates COMPGN1F (Thermo Trek Diagnostics, Waltham, MA, USA). The susceptibility of the isolates was assessed against 20 antimicrobials of different groups (aminoglycosides: amikacin and gentamicin; aminopenicillin: ampicillin; β-lactam/β-lactamase inhibitor: amoxicillin/clavulanic acid and piperacillin/tazobactam; cephalosporin I: cefalexin, cefazolin; cephalosporin III: cefovecin, cefpodoxime, and ceftazidime; fluoroquinolone: enrofloxacin, marbofloxacin, orbifloxacin, and pradofloxacin; polymyxins: colistin; folate pathway inhibitors: trimethoprim/sulfamethoxazole; phenicols: chloramphenicol; tetracyclines: doxycycline and tetracycline; and carbapenem: imipenem). *E. coli* ATCC 25922 was used as a quality control strain. The minimum inhibitory concentrations (MICs) were interpreted according to the guidelines provided by the Clinical and Laboratory Standard Institute [57]. The lowest antimicrobial concentrations, at which the growth of the isolates was inhibited by 50% and 90%, were denoted as MIC_50_ and MIC_90_, respectively. Multidrug-resistant isolates demonstrate resistance to a minimum of three distinct categories of antimicrobial agents [58].

### 4.3. Statistical Analysis

Antimicrobial resistance rates and Pearson correlation were analyzed using Microsoft Excel 2016 (Microsoft Corporation, Redmond, WA, USA) and Rex Software (version 3.0.3, RexSoft Inc., Seoul, Republic of Korea). The chi-square test was used to compare the observed resistance rates. *p* values < 0.05 were considered statistically significant.

## 5. Conclusions

The findings of our investigation indicated that the *E. coli* isolates obtained from healthy dogs and cats exhibited resistance to several antimicrobials, including those considered critically important for use in humans. Among them, cefalexin resistance was highly detected, followed by ampicillin, tetracycline, and cefazolin resistance. Moreover, MDR in isolates from dogs and cats was significantly observed, and often, the MDR patterns encompassed more than five antimicrobials. The potential cross-transmission of resistant bacteria between companion animals and humans presents a significant challenge within the field of humans and veterinary medicine. This challenge is extending concerns related to the broader public health perspective day by day. Therefore, comprehensive surveillance is of utmost importance to understand the transmission mechanisms of antimicrobial resistance between companion animals and humans and reduce the possible hazards of *E. coli*.

## Figures and Tables

**Table 1:** Antimicrobial resistance of *Escherichia coli* isolated from dogs and cats during 2020–2022 in Korea.

	% (Resistant Isolates)			
Antimicrobials	Dogs (*n* = 637)	Cats (*n* = 206)	Total (*n* = 843)	*p*-Value
Amikacin	0.6 (4)	0 (0)	0.5 (4)	0.2547
Amoxicillin/clavulanic acid	7.2 (46)	3.9 (8)	6.4 (54)	0.0891
Ampicillin	42.4 (270)	25.7 (53)	38.3 (323)	<0.0001
Cefalexin	74.4 (474)	51.9 (107)	68.9 (581)	<0.0001
Cefazolin	20.4 (130)	13.6 (28)	18.7 (158)	0.0293
Cefovecin	16.2 (103)	9.7 (20)	14.6 (123)	0.0223
Cefpodoxime	16.5 (105)	9.7 (20)	14.8 (125)	0.0173
Ceftazidime	6.0 (38)	4.9 (10)	5.7 (48)	0.5502
Chloramphenicol	13.2 (84)	7.8 (16)	11.9 (100)	0.0365
Colistin	0.2 (1)	0 (0)	0.1 (1)	0.5698
Doxycycline	15.9 (101)	11.7 (24)	14.8 (125)	0.1401
Enrofloxacin	16.2 (103)	6.3 (13)	13.8 (116)	0.0003
Gentamicin	8.3 (53)	4.4 (9)	7.4 (62)	0.0590
Imipenem	0.2 (1)	0 (0)	0.1 (1)	0.5698
Marbofloxacin	16.2 (103)	6.3 (13)	13.8 (116)	0.0003
Orbifloxacin	17.4 (111)	7.3 (15)	14.9 (126)	0.0003
Piperacillin/tazobactam	0.6 (4)	0 (0)	0.5 (4)	0.2547
Pradofloxacin	15.7 (100)	6.3 (13)	13.4 (113)	0.0005
Tetracycline	25.6 (163)	15.5 (32)	23.1 (195)	0.0028
Trimethoprim/sulfamethoxazole	17.3 (110)	6.3 (13)	14.6 (123)	0.0001
MDR	34.9 (222)	20.9 (43)	42.3 (357)	<0.0001

*p* < 0.05 was considered to indicate a significant change in antimicrobial resistance rate. MDR, multidrug resistance.

**Table 2:** Distribution of antimicrobial-resistant *Escherichia coli* among different age groups of dogs and cats isolated during 2020–2022 in South Korea.

Antimicrobials	Resistance % (Isolates)												
	Dogs (*n* = 637)							Cats (*n* = 206)					
	1 Year: Puppy and Juvenile (*n* = 146)	2–5 Years: Mature Adult (*n* = 196)	6–10 Years: Senior (*n* = 206)	≥11 Years: Geriatric (*n* = 87)	Unknown (*n* = 2)	Total	*p*-Value	≤1 Year: Kitten (*n* = 75)	2–6 Years: Junior and Adults (*n* = 98)	7–10 Years: Mature (*n* = 26)	≥11 Years: Senior (*n* = 7)	Total	*p*-Value
Amikacin	0.7 (1)	0.5 (1)	0 (0)	2.3 (2)	0 (0)	0.6 (4)	0.2629	0 (0)	0 (0)	0 (0)	0 (0)	0 (0)	–
Amoxicillin/clavulanic acid	6.8 (10)	4.1 (8)	8.7 (18)	11.5 (10)	0 (0)	7.2 (46)	0.1890	1.3 (1)	5.1 (5)	7.7 (2)	0 (0)	3.9 (8)	0.3500
Ampicillin	44.5 (65)	40.8 (80)	43.2 (89)	41. 4 (36)	0 (0)	42.4 (270)	0.7312	25.3 (19)	25.5 (25)	34.6 (9)	0 (0)	25.7 (53)	0.3236
Cefalexin	79.5 (116)	69.4 (136)	77.7 (160)	71.3 (62)	0 (0)	74.4 (474)	0.0173	50.7 (38)	56.1 (55)	46.2 (12)	28.6 (2)	51.9 (107)	0.4596
Cefazolin	20.5 (30)	16.8 (33)	24.3 (50)	19.5 (17)	0 (0)	20.4 (130)	0.4094	8.0 (6)	16.3 (16)	23.1 (6)	0 (0)	14.6 (30)	0.1273
Cefovecin	15.1 (22)	14.3 (28)	18.9 (39)	16.1 (14)	0 (0)	16.2 (103)	0.7027	6.7 (5)	10.2 (10)	19.2 (5)	0 (0)	9.7 (20)	0.2374
Cefpodoxime	15.1 (22)	14.3 (28)	19.9 (41)	16.1 (14)	0 (0)	16.5 (105)	0.5507	6.7 (5)	10.2 (10)	19.2 (5)	0 (0)	9.7 (20)	0.2374
Ceftazidime	8.2 (12)	5.1 (10)	6.3 (13)	3.4 (3)	0 (0)	6.0 (38)	0.6049	4.0 (3)	4.1 (4)	11.5 (3)	0 (0)	4.9 (10)	0.3782
Chloramphenicol	16.4 (24)	13.8 (27)	11.2 (23)	10.3 (9)	50.0 (1)	13.2 (84)	0.2758	8.0 (6)	7.1 (7)	11.5 (3)	0 (0)	7.8 (16)	0.7654
Colistin	0.7 (1)	0 (0)	0 (0)	0 (0)	0 (0)	0.2 (1)	0.5001	0 (0)	0 (0)	0 (0)	0 (0)	0 (0)	–
Doxycycline	22.6 (33)	15.3 (30)	13.6 (28)	11.5 (10)	0 (0)	15.9 (101)	0.1147	13.3 (10)	12.2 (12)	7.7 (2)	0 (0)	11.7 (24)	0.6734
Enrofloxacin	13.7 (20)	12.8 (25)	18.0 (37)	24.1 (21)	0 (0)	16.2 (103)	0.1214	9.3 (7)	4.1 (4)	7.7 (2)	0 (0)	6.3 (13)	0.4734
Gentamicin	8.2 (12)	4.1 (8)	9.7 (20)	14.9 (13)	0 (0)	8.3 (53)	0.0350	6.7 (5)	1.0 (1)	11.5 (3)	0 (0)	4.4 (9)	0.0688
Imipenem	0 (0)	0 (0)	0 (0)	1.1 (1)	0 (0)	0.2 (1)	0.1762	0 (0)	0 (0)	0 (0)	0 (0)	0 (0)	–
Marbofloxacin	13.7 (20)	12.8 (25)	18.0 (37)	24.1 (21)	0 (0)	16.2 (103)	0.1214	9.3 (7)	4.1 (4)	7.7 (2)	0 (0)	6.3 (13)	0.4734
Orbifloxacin	15.1 (22)	13.3 (26)	19.4 (40)	26.4 (23)	0 (0)	17.4 (111)	0.0655	10.7 (8)	5.1 (5)	7.7 (2)	0 (0)	7.3 (15)	0.4768
Piperacillin/tazobactam	0 (0)	1.0 (2)	0.5 (1)	1.1 (1)	0 (0)	0.6 (4)	0.7621	0 (0)	0 (0)	0 (0)	0 (0)	0 (0)	–
Pradofloxacin	13.7 (20)	12.2 (24)	18.0 (37)	21.8 (19)	0 (0)	15.7 (100)	0.2109	9.3 (7)	4.1 (4)	7.7 (2)	0 (0)	6.3 (13)	0.4734
Tetracycline	30.1 (44)	27.6 (54)	22.3 (46)	21.8 (19)	0 (0)	25.6 (163)	0.3486	20.0 (15)	14.3 (14)	11.5 (3)	0 (0)	15.5 (32)	0.4183
Trimethoprim/sulfamethoxazole	23.3 (34)	17.3 (34)	15.0 (31)	12.6 (11)	0 (0)	17.3 (110)	0.1900	12.0 (9)	3.1 (3)	3.8 (1)	0 (0)	6.3 (13)	0.0862
MDR	36.3 (53)	32.1 (63)	37.9 (78)	32.2 (28)	0 (0)	34.9 (222)	0.5708	18.7 (14)	20.4 (20)	34.6 (9)	0 (0)	20.9 (43)	0.1696

*p* < 0.05 was considered to indicate a significant change in antimicrobial resistance rate. MDR, multidrug resistance.

**Table 3:** Frequent resistance patterns in *Escherichia coli* isolated from dogs (*n* = 637) during 2020–2022 in South Korea.

No. of Antimicrobials	% (No. of Resistant Isolates)	Most Common Pattern (No. of Isolates)
No resistance	14.9% (95)	–
Resistance 1 agent	34.7% (221)	LEX (*n* = 203)
Resistance 2 agents	9.3% (59)	AMP LEX (*n* = 34)
Resistance 3 agents	6.4% (41)	LEX DOX TET (*n* = 10)
Resistance 4 agents	5.5% (35)	AMP LEX DOX TET (*n* = 7)
Resistance 5 agents	7.2% (46)	AMP LEX CFZ VEC CPD (*n* = 14)
Resistance 6 agents	5.0% (32)	AMC AMP LEX CFZ VEC CPD (*n* = 8)
Resistance 7 agents	3.5% (22)	AMC AMP LEX CFZ VEC CPD CAZ (*n* = 6)
Resistance 8 agents	2.2% (14)	AMC AMP LEX CFZ VEC CPD CAZ SXT (*n* = 2) AMP LEX CHL ENO MAR ORB PRA TET (*n* = 2)
Resistance 9 agents	3.6% (23)	AMP LEX CFZ VEC CPD ENO MAR ORB PRA (*n* = 5)
Resistance 10 agents	1.7% (11)	AMP LEX CFZ VEC CPD ENO MAR ORB PRA SXT (*n* = 4)
Resistance 11 agents	0.6% (4)	AMC AMP LEX CFZ VEC CPD CAZ ENO MAR ORB PRA (*n* = 2)
Resistance 12 agents	2.0% (13)	AMP LEX CFZ VEC CPD DOX ENO MAR ORB PRA TET SXT (*n* = 3)
Resistance 13 agents	0.8% (5)	AMP LEX CFZ VEC CPD CHL DOX ENO MAR ORB PRA TET SXT (*n* = 2)
Resistance 14 agents	1.3% (8)	AMP LEX CFZ VEC CPD CAZ CHL DOX ENO MAR ORB PRA TET SXT (*n* = 3)
Resistance 15 agents	1.3% (8)	AMC AMP LEX CFZ VEC CPD CAZ CHL DOX ENO MAR ORB PRA TET SXT (*n* = 4)

AMC, amoxicillin/clavulanic acid; AMP, ampicillin; CAZ, ceftazidime; CFZ, cefazolin; CHL, chloramphenicol; CPD, cefpodoxime, DOX, doxycycline; ENO, enrofloxacin; LEX, cefalexin; MAR, marbofloxacin; ORB, orbifloxacin; PRA, pradofloxacin; SXT, trimethoprim/sulphamethoxazole; TET, tetracycline; VEC, cefovecin.

**Table 4:** Frequent resistance patterns in *Escherichia coli* isolated from cats (*n* = 206) during 2020–2022 in South Korea.

No. of Antimicrobials	% (No. of Resistant Isolates)	Most Common Pattern (No. of Isolates)
No resistance	42.2% (87)	–
Resistance 1 agent	29.6% (61)	LEX (*n* = 58)
Resistance 2 agents	2.9% (6)	AMP LEX (*n* = 4)
Resistance 3 agents	5.8% (12)	AMP LEX CFZ (*n* = 3)
Resistance 4 agents	3.4% (7)	AMP LEX DOX TET (*n* = 4)
Resistance 5 agents	3.9% (8)	AMP LEX CFZ VEC CPD (*n* = 2) AMP LEX CHL DOX TET (*n* = 2)
Resistance 6 agents	2.9% (6)	AMP LEX CFZ CHL DOX TET (*n* = 3)
Resistance 7 agents	2.9% (6)	AMC AMP LEX CFZ VEC CPD CAZ (*n* = 4)
Resistance 8 agents	1.0% (2)	AMC AMP LEX CFZ VEC CPD DOX TET (*n* = 1) AMP LEX CFZ VEC CPD GEN TET SXT (*n* = 1)
Resistance 9 agents	1.5% (3)	AMP LEX CFZ VEC CPD ENO MAR ORB PRA (*n* = 2)
Resistance 10 agents	1.0% (2)	AMP LEX DOX ENO GEN MAR ORB PRA TET SXT (*n* = 1) AMP LEX CFZ VEC CPD CAZ DOX ORB TET SXT (*n* = 1)
Resistance 11 agents	1.0% (2)	AMP LEX CFZ VEC CPD CAZ ENO GEN MAR ORB PRA (*n* = 1) AMP LEX CFZ VEC CPD DOX ENO MAR ORB PRA TET (*n* = 1)
Resistance 12 agents	0.5% (1)	AMP LEX CFZ VEC CPD CHL DOX ENO MAR ORB PRA TET (*n* = 1)
Resistance 14 agents	1.5% (3)	AMP LEX CFZ VEC CPD CAZ DOX ENO GEN MAR ORB PRA TET SXT (*n* = 2)

AMC, amoxicillin/clavulanic acid; AMP, ampicillin; CAZ, ceftazidime; CFZ, cefazolin; CHL, chloramphenicol; CPD, cefpodoxime, DOX, doxycycline; ENO, enrofloxacin; GEN, gentamicin; LEX, cefalexin; MAR, marbofloxacin; ORB, orbifloxacin; PRA, pradofloxacin; SXT, trimethoprim/sulphamethoxazole; TET, tetracycline; VEC, cefovecin.

**Table 5:** *Escherichia coli* isolates recovered from dogs and cats during 2020–2022 in South Korea.

Year	Dogs		Cats	
	Animal Hospitals	Isolates	Animal Hospitals	Isolates
2020	22	193	16	67
2021	25	181	15	71
2022	27	263	20	68
Total	74	637	51	206

## Data Availability

All data generated for this study are contained within the article/Supplementary Materials.

## Supplementary Materials

The following supporting information can be downloaded at https://www.mdpi.com/article/10.3390/antibiotics13010027/s1, Table S1: The MIC_50_ and MIC_90_ of the tested antimicrobials against *Escherichia coli* isolated from dogs (*n* = 637) and cats (*n* = 206) during 2020–2022 in South Korea; Table S2: Antimicrobial resistance rate in *Escherichia coli* isolated from dogs (*n* = 637) and cats (*n* = 206) during 2020–2022 in South Korea; Table S3: Antimicrobial resistance patterns of *Escherichia coli* isolated from dogs (*n* = 637) during 2020–2022 in South Korea; Table S4: Antimicrobial resistance patterns of *Escherichia coli* isolated from cats (*n* = 206) during 2020–2022 in South Korea.
