# Supplementary material for: Antimicrobial Resistance in Escherichia coli Isolated from Healthy Dogs and Cats in South Korea, 2020–2022
Source: Antibiotics (Basel). 2023 Dec 27;13(1):27. doi: 10.3390/antibiotics13010027 (PMC10812631; doi:10.3390/antibiotics13010027)
Supplement: Supplementary file 1 [file antibiotics-13-00027-s001.zip › antibiotics-2736825-supplementary.pdf]

Table S1. The MIC_{50} and MIC_{90} of the tested antimicrobials against *Escherichia coli* isolated from dogs (n = 637) and cats (n = 206) during 2020–2022 in South Korea

Antimicrobials	Dogs (n = 637)				Cats (n = 206)			
	2020 (n = 193)	2021 (n = 181)	2022 (n = 263)	Total (n = 637)	2020 (n = 67)	2021 (n = 71)	2022 (n = 68)	Total (n = 206)
Amikacin								
MIC_{50}	4	4	4	4	4	4	4	4
MIC_{90}	4	4	4	4	4	4	4	4
% Resistance (No. of isolates)	0 (0)	0.6 (1)	1.1 (3)	0.4 (4)	0 (0)	0 (0)	0 (0)	0 (0)
Amoxicillin/ clavulanic acid								
MIC_{50}	4	4	4	8	4	4	4	4
MIC_{90}	16	8	8	16	8	8	8	8
% Resistance (No. of isolates)	6.7 (13)	4.4 (8)	9.5 (25)	8.2 (73)	0 (0)	7 (5)	4.4 (3)	5.5 (17)
Ampicillin								
MIC_{50}	8	4	8	8	4	4	8	4
MIC_{90}	64	64	64	64	64	64	64	64
% Resistance (No. of isolates)	43 (83)	35.9 (65)	46.4 (122)	44 (393)	25.4 (17)	31 (22)	20.6 (14)	28.4 (88)
Cefalexin								
MIC_{50}	8	8	8	8	8	8	4	8
MIC_{90}	32	16	8	16	32	16	16	16
% Resistance (No. of isolates)	80.3 (155)	77.9 (141)	67.7 (178)	53.1 (474)	56.7 (38)	60.6 (43)	38.2 (26)	34.5 (107)
Cefazolin								
MIC_{50}	2	2	2	4	2	2	2	2
MIC_{90}	32	32	4	32	8	8	32	32
% Resistance (No. of isolates)	18.1 (35)	14.4 (26)	26.2 (69)	23.1 (206)	11.9 (8)	15.5 (11)	13.2 (9)	14.5 (45)
Cefovecin								
MIC_{50}	1	1	1	1	0.5	1	0.5	0.5
MIC_{90}	8	8	1	8	2	2	8	8
% Resistance (No. of isolates)	14 (27)	12.2 (22)	20.5 (54)	19 (170)	9 (6)	9.9 (7)	10.3 (7)	11.3 (35)
Cefpodoxime								
MIC_{50}	1	1	1	1	1	1	1	1
MIC_{90}	8	8	1	8	4	1	8	8

% Resistance (No. of isolates)	14.5 (28)	12.2 (22)	20.9 (55)	19.3 (172)	9 (6)	9.9 (7)	10.3 (7)	11.3 (35)
Ceftazidime								
MIC_{50}	4	4	4	4	4	4	4	4
MIC_{90}	4	4	4	4	4	4	4	4
% Resistance (No. of isolates)	5.7 (11)	3.3 (6)	8 (21)	4.3 (38)	3 (2)	7 (5)	4.4 (3)	3.2 (10)
Chloramphenicol								
MIC_{50}	8	8	8	8	8	8	8	8
MIC_{90}	32	16	8	32	8	16	32	16
% Resistance (No. of isolates)	13.5 (26)	9.9 (18)	15.2 (40)	13.4 (120)	3 (2)	8.5 (6)	11.8 (8)	10 (31)
Doxycycline								
MIC_{50}	2	2	2	2	1	2	1	8
MIC_{90}	16	16	2	16	16	16	4	16
% Resistance (No. of isolates)	18.7 (36)	12.7 (23)	16 (42)	16.1 (144)	16.4 (11)	14.1 (10)	4.4 (3)	11.3 (35)
Enrofloxacin								
MIC_{50}	0.12	0.12	0.12	0.25	0.12	0.12	0.12	0.12
MIC_{90}	4	2	0.25	4	4	0.5	1	1
% Resistance (No. of isolates)	16.1 (31)	9.9 (18)	20.5 (54)	17.6 (157)	10.4 (7)	0 (0)	8.8 (6)	9 (28)
Gentamicin								
MIC_{50}	0.5	0.5	0.5	0.5	0.5	0.5	0.5	0.5
MIC_{90}	8	1	0.5	16	1	1	1	1
% Resistance (No. of isolates)	9.8 (19)	7.7 (14)	7.6 (20)	10.8 (96)	7.5 (5)	1.4 (1)	4.4 (3)	5.8 (18)
Imipenem								
MIC_{50}	1	1	1	1	1	1	1	1
MIC_{90}	1	1	1	1	1	1	1	1
% Resistance (No. of isolates)	0.5 (1)	0 (0)	0 (0)	0.1 (1)	0 (0)	0 (0)	0 (0)	0 (0)
Marbofloxacin								
MIC_{50}	0.12	0.12	0.12	0.25	0.12	0.12	0.12	0.12
MIC_{90}	4	2	0.25	4	4	0.5	1	1
% Resistance (No. of isolates)	16.1 (31)	9.9 (18)	20.5 (54)	17.6 (157)	10.4 (7)	0 (0)	8.8 (6)	9 (28)
Orbifloxacin								
MIC_{50}	1	1	1	1	1	1	1	1
MIC_{90}	8	8	1	8	8	1	4	2
% Resistance (No. of isolates)	16.6 (32)	11 (20)	22.4 (59)	12.4 (111)	11.9 (8)	1.4 (1)	8.8 (6)	4.8 (15)

Piperacillin/ tazobactam								
MIC$_{50}$	8	8	8	8	8	8	8	8
MIC$_{90}$	8	8	8	8	8	8	8	8
% Resistance (No. of isolates)	1 (2)	0 (0)	0.8 (2)	0.4 (4)	0 (0)	0 (0)	0 (0)	0 (0)
Paradofloxacin								
MIC$_{50}$	0.25	0.25	0.25	0.25	0.25	0.25	0.25	0.25
MIC$_{90}$	2	1	0.25	2	2	0.25	0.25	0.25
% Resistance (No. of isolates)	15 (29)	9.9 (18)	20.2 (53)	11.2 (100)	10.4 (7)	0 (0)	8.8 (6)	4.2 (13)
Tetracycline								
MIC$_{50}$	4	4	4	4	4	4	4	4
MIC$_{90}$	16	16	4	16	16	16	16	16
% Resistance (No. of isolates)	29.5 (57)	24.3 (44)	23.6 (62)	18.3 (163)	17.9 (12)	18.3 (13)	10.3 (7)	10.3 (32)
Trimethoprim/ sulfamethoxazole								
MIC$_{50}$	0.5	0.5	0.5	0.5	0.5	0.5	0.5	0.5
MIC$_{90}$	4	4	0.5	4	0.5	0.5	0.5	2
% Resistance (No. of isolates)	18.7 (36)	13.3 (24)	19 (50)	19.5 (174)	7.5 (5)	4.2 (3)	7.4 (5)	9.7 (30)
Colistin								
MIC$_{50}$	1	1	1	1	1	1	1	1
MIC$_{90}$	1	1	1	1	1	1	1	1
% Resistance (No. of isolates)	0 (0)	0 (0)	0.4 (1)	0.1 (1)	0 (0)	0 (0)	0 (0)	0 (0)
MDR	36.3 (70)	28.7 (52)	38 (100)	35.2 (314)	17.9 (12)	23.9 (17)	20.6 (14)	20.3 (63)

MIC$_{50}$ and MIC$_{90}$ are the concentrations (μg/mL) at which 50% and 90% of the isolates were inhibited, respectively.

Table S2. Antimicrobial resistance rate in *Escherichia coli* isolated from dogs (n = 637) and cats (n = 206) during 2020–2022 in South Korea

Antimicrobials	Resistance rate % (isolates)									
	Dogs (n = 637)					Cats (n = 206)				
	2020 (n = 193)	2021 (n = 181)	2022 (n = 263)	Subtotal (n = 637)	*p*-value	2020 (n = 67)	2021 (n = 71)	2022 (n = 68)	Subtotal (n = 206)	*p*-value
Amikacin	0 (0)	0.6 (1)	1.1 (3)	0.6 (4)	0.3107	0 (0)	0 (0)	0 (0)	0 (0)	–

Amoxicillin/clavulanic acid	6.7 (13)	4.4 (8)	9.5 (25)	7.2 (46)	0.1205	0 (0)	7.0 (5)	4.4 (3)	3.9 (8)	0.0982
Ampicillin	43.0 (83)	35.9 (65)	46.4 (122)	42.4 (270)	0.0881	25.4 (17)	31.0 (22)	20.6 (14)	25.7 (53)	0.3767
Cefalexin	80.3 (155)	77.9 (141)	67.7 (178)	74.4 (474)	0.0041	56.7 (38)	60.6 (43)	38.2 (26)	51.9 (107)	0.0194
Cefazolin	18.1 (35)	14.4 (26)	26.2 (69)	20.4 (130)	0.0060	11.9 (8)	15.5 (11)	13.2 (9)	13.6 (28)	0.8285
Cefovecin	14.0 (27)	12.2 (22)	20.5 (54)	16.2 (103)	0.0382	9.0 (6)	9.9 (7)	10.3 (7)	9.7 (20)	0.9652
Cefpodoxime	14.5 (28)	12.2 (22)	20.9 (55)	16.5 (105)	0.0340	9.0 (6)	9.9 (7)	10.3 (7)	9.7 (20)	0.9652
Ceftazidime	5.7 (11)	3.3 (6)	8.0 (21)	6.0 (38)	0.1226	3.0 (2)	7 (5)	4.4 (3)	4.9 (10)	0.5334
Chloramphenicol	13.5 (26)	9.9 (18)	15.2 (40)	13.2 (84)	0.2714	3.0 (2)	8.5 (6)	11.8 (8)	7.8 (16)	0.1587
Colistin	0 (0)	0 (0)	0.4 (1)	0.2 (1)	0.4918	0 (0)	0 (0)	0 (0)	0 (0)	–
Doxycycline	18.7 (36)	12.7 (23)	16.0 (42)	15.9 (101)	0.2904	16.4 (11)	14.1 (10)	4.4 (3)	11.7 (24)	0.0692
Enrofloxacin	16.1 (31)	9.9 (18)	20.5 (54)	16.2 (103)	0.0117	10.4 (7)	0 (0)	8.8 (6)	6.3 (13)	0.0238
Gentamicin	9.8 (19)	7.7 (14)	7.6 (20)	8.3 (53)	0.6562	7.5 (5)	1.4 (1)	4.4 (3)	4.4 (9)	0.2228
Imipenem	0.5 (1)	0 (0)	0 (0)	0.2 (1)	0.3170	0 (0)	0 (0)	0 (0)	0 (0)	–
Marbofloxacin	16.1 (31)	9.9 (18)	20.5 (54)	16.2 (103)	0.0117	10.4 (7)	0 (0)	8.8 (6)	6.3 (13)	0.0238
Orbifloxacin	16.6 (32)	11.0 (20)	22.4 (59)	17.4 (111)	0.0073	11.9 (8)	1.4 (1)	8.8 (6)	7.3 (15)	0.0492
Piperacillin/tazobactam	1.0 (2)	0 (0)	0.8 (2)	0.6 (4)	0.4215	0 (0)	0 (0)	0 (0)	0 (0)	–
Paradofloxacin	15 (29)	9.9 (18)	20.2 (53)	15.7 (100)	0.0138	10.4 (7)	0 (0)	8.8 (6)	6.3 (13)	0.0238
Tetracycline	29.5 (57)	24.3 (44)	23.6 (62)	25.6 (163)	0.3187	17.9 (12)	18.3 (13)	10.3 (7)	15.5 (32)	0.3485
Trimethoprim/sulfamethoxazole	18.7 (36)	13.3 (24)	19.0 (50)	17.3 (110)	0.2408	7.5 (5)	4.2 (3)	7.4 (5)	6.3 (13)	0.6745
MDR	36.3(70)	28.7 (52)	38 (100)	34.9 (222)	0.11543	17.9 (12)	23.9 (17)	20.6 (14)	20.9 (43/209)	0.6855

$p < 0.05$ was considered a significant change in the antimicrobial resistance trend. MDR, multidrug resistant.

Table S3. Antimicrobial resistance patterns of *Escherichia coli* isolated from dogs (n = 637) during 2020–2022 in South Korea

Antimicrobials (isolates)	Resistance patterns (isolates)
0 (n = 95)	–
1 (n = 221)	AMP(n=11), CHL(n=1), GEN(n=2), LEX(n=203), ORB(n=1), SXT(n=1), TET(n=2)
2 (n = 59)	AMP CFZ(n=1), AMP CHL(n=1), AMP LEX(n=34), AMP SXT(n=2), AMP TET(n=2), CHL SXT(n=1), DOX TET(n=4), LEX CHL(n=6), LEX GEN(n=1), LEX ORB(n=2), LEX TET(n=3), TET SXT(n=2)
3 (n = 41)	AMC AMP LEX(n=1), AMP CHL TET(n=2), AMP DOX TET(n=2), AMP LEX CFZ(n=5), AMP LEX GEN(n=2), AMP LEX SXT(n=6), AMO LEX TET(n=6), AMP TET SXT(n=1), DOX TET SXT(n=1), ENO MAR ORB(n=1), GEN TET SXT(n=1), LEX CHL TET(n=1), LEX DOX TET(n=10), LEX GEN TET(n=1), LEX TET SXT(n=1)
4 (n = 35)	AMC AMP LEX CFZ(n=1), AMP CHL DOX TET(n=1), AMP CHL GEN SXT(n=1), AMP CHL ORB SXT(n=1), AMP CHL TET SXT(n=1), AMP DOX TET SXT(n=2), AMO LEX CFZ CHL(n=1), AMP LEX CFZ GEN(n=1), AMP LEX CFZ SXT(n=2), AMP LEX CFZ TET(n=1), AMP LEX CHL TET(n=2), AMP LEX DOX TET(n=7), AMP LEX GEN TET(n=1), AMP LEX TET SXT(n=3), ENO MAR ORB PRA(n=4), LEX CFZ VEC CHL(n=1), LEX CHL DOX TET(n=3), LEX CHL TET SXT(n=1), LEX DOX TET SXT(n=1)
5 (n = 46)	AMC AMP LEX CFZ CPD(n=1), AMC AMP LEX CFZ PTZ(n=1), AMP CHL DOX ORB TET(n=1), AMP ENO MAR ORB PRA(n=4), AMP LEX CFZ DOX TET(n=2), AMP LEX CFZ VEC CPD(n=14), AMP LEX CHL DOX TET(n=2), AMP LEX CHL TET SXT(n=2), AMP LEX DOX TET SXT(n=2), AMP LEX GEN TET SXT(n=2), AMP LEX ORB TET SXT(n=1), CHL ENO MAR ORB PRA(n=1), LEX CHL DOX GEN TET(n=1), LEX CHL DOX TET SXT(n=1), LEX ENO MAR ORB PRA(n=11)
6 (n = 32)	AMC AMP LEX CFZ CHL PTZ(n=1), AMC AMP LEX CFZ VEC CPD(n=8), AMP CHL DOX GEN TET SXT(n=2), AMP LEX CFZ CHL TET SXT(n=1), AMP LEX CFZ DOX TET SXT(n=1), AMP LEX CFZ VEC CPD CAZ(n=4), AMP LEX CFZ VEC CPD GEN(n=1), AMP LEX CFZ VEC CPD TET(n=1), AMP LEX CHL DOX TET SXT(n=5), AMP LEX DOX GEN ORB TET(n=2), AMP LEX DOX GEN TET SXT(n=3), AMP LEX ENO MAR PRB PRA(n=2), DOX ENO MAR ORB PRA TET(n=1)
7 (n = 22)	AMC AMP LEX CFZ VEC CPD CAZ(n=6), AMC AMP LEX CFZ VEC CPD GEN(n=2), AMP CHL ENO GEN MAR ORB PRA(n=1), AMP CHL ENO MAR ORB PRA TET(n=1), AMP ENO GEN MAR ORB PRA SXT(n=2), AMP ENO GEN MAR ORB PRA TET(n=1), AMP LEX CFZ DOX GEN TET SXT(n=1), AMP LEX CFZ ENO MAR ORB PRA(n=1), AMO LEX CFZ VEC CPD CAZ COL(n=1), AMP LEX CFZ VEC CPD CAZ SXT(n=1), AMP LEX CFZ VEC CPD CAZ TET(n=2), AMP LEX ENO MAR ORB PRA SXT(n=2), LEX CHL ENO MAR ORB PRA TET(n=1)
8 (n = 14)	AMC AMP LEX CFZ VEC CPD CAZ SXT(n=2), AMC AMP LEX CFZ VEC CPD CAZ TET(n=1), AMC AMP LEX CFZ VEC CPD DOX TET(n=1), AMK AMP LEX CFZ DOX GEN TET SXT(n=1), AMP DOX ENO GEN MAR ORB PRA TET(n=1), AMP DOX ENO MAR ORB PRA TET SXT(n=1), AMP LEX CFZ VEC CPD CAZ CHL TET(n=1), AMP LEX CFZ VEC CPD CHL DOX TET(n=1), AMP LEX CFZ VEC CPD DOX TET SXT(n=1), AMP LEX CHL ENO MAR ORB PRA TET(n=2), AMP LEX ENO MAR ORB PRA TET SXT(n=1), LEX DOX ENO MAR ORB PRA TET SXT(n=1)

9 (n = 23)	AMC AMP CHL ENO MAR ORB PRA TET SXT(n=1), AMC AMP LEX CFZ VEC CPD CAZ PTZ SXT(n=1), AMP CHL DOX ENO MAR ORB PRA TET SXT(n=1), AMP CHL ENO GEN MAR ORB PRA TET SXT(n=1), AMP LEX CFZ ENO MAR ORB PRA TET SXT(n=2), AMP LEX CFZ VEC CPD CHL ENO MAR ORB(n=1), AMP LEX CFZ VEC CPD DOX GEN TET SXT(n=2), AMP LEX CFZ VEC CPD ENO MAR ORB PRA(n=5), AMP LEX CHL DOX ENO MAR ORB PRA TET(n=3), AMP LEX CHL ENO GEN MAR ORB PRA SXT(n=1), AMP LEX CHL ENO MAR ORB PRA TET SXT(n=1), AMP LEX DOX ENO MAR ORB PRA TET SXT(n=3), AMP LEX ENO GEN MAR ORB PRA TET SXT(n=1)
10 (n = 11)	AMC AMP LEX CFZ CPD ENO GEN MAR ORB PRA(n=1), AMC AMP LEX CFZ VEC CPD CAZ CHL TET SXT(n=1), AMC AMP LEX CFZ VEC CPD CHL DOX TET SXT(n=1), AMP LEX CFZ CHL DOX ENO MAR ORB PRA TET(n=1), AMP LEX CFZ VEC CPD CAZ CHL DOX TET SXT(n=1), AMP LEX CFZ VEC CPD CHL ENO MAR ORB PRA(n=1), AMP LEX CFZ VEC CPD ENO GEN MAR ORB PRA(n=1), AMP LEX CFZ VEC CPD ENO MAR ORB PRA SXT(n=4)
11 (n = 4)	AMC AMP LEX CFZ VEC CPD CAZ ENO MAR ORB PRA (n=2), AMC AMP LEX CFZ VEC CPD CHL ENO MAR ORB PRA(n=1), AMK AMP LEX CFZ VEC CPD CHL DOX GEN TET SXT(n=1)
12 (n = 13)	AMC AMP LEX CFZ CPD DOX ENO GEN MAR ORB PRA TET(n=1), AMC AMP LEX CFZ VEC CPD CAZ DOX IPM PTZ TET SXT(n=1), AMC AMP LEX CFZ VEC CPD ENO GEN MAR ORB PRA SXT(n=1), AMP LEX CFZ VEC CPD CAZ CHL ENO MAR ORB PRA TET(n=1), AMP LEX CFZ VEC CPD CAZ DOX ENO GEN MAR ORB TET(n=1), AMP LEX CFZ VEC CPD CHL DOX ENO MAR ORB PRA TET(n=2), AMP LEX CFZ VEC CPD CHL ENO MAR ORB PRA TET SXT(n=2), AMP LEX CFZ VEC CPD DOX ENO MAR ORB PRA TET SXT(n=3), AMP LEX CFZ VEC CPD ENO GEN MAR ORB PRA TET SXT(n=1)
13 (n = 5)	AMK AMP LEX CFZ VEC CPD CHL ENO GEN MAR ORB PRA TET(n=1), AMP LEX CFZ VEC CPD CAZ ENO GEN MAR ORB PRA TET SXT(n=1), AMP LEX CFZ VEC CPD CHL DOX ENO MAR ORB PRA TET SXT(n=2), AMP LEX CFZ VEC CPD DOX ENO GEN MAR ORB PRA TET SXT(n=1)
14 (n = 8)	AMC AMP LEX CFZ VEC CPD CAZ CHL DOX ENO MAR ORB PRA TET(n=1), AMC AMP LEX CFZ VEC CPD CAZ ENO GEN MAR ORB PRA TET SXT(n=1), AMC AMP LEX CFZ VEC CPD DOX ENO GEN MAR ORB PRA TET SXT(n=2), AMP LEX CFZ VEC CPD CAZ CHL DOX ENO MAR ORV PRA TET SXT(n=3), AMP LEX CFZ VEC CPD CHL DOX ENO GEN MAR ORB PRA TET SXT(n=1)
15 (n = 8)	AMC AMP LEX CFZ VEC CPD CAZ CHL DOX ENO MAR ORB PRA TET SXT(n=4), AMC AMP LEX CFZ VEC CPD CAZ DOX ENO GEN MAR ORB PRA TET SXT(n=2), AMC AMP LEX CFZ VEC CPD CHL DOX ENO GEN MAR ORB PRA TET SXT(n=1), AMP LEX CFZ VEC CPD CAZ CHL DOX ENO GEN MAR ORB PRA TET SXT(n=1)

AMC, amoxicillin; AMK, amikacin/clavulanic acid; AMP, ampicillin; CAZ, ceftazidime; CFZ, cefazolin; CHL, chloramphenicol; COL, colistin; CPD, cefpodoxime, DOX, doxycycline; ENO, enrofloxacin; GEN, gentamicin; IPM, imipenem; LEX, cefalexin; MAR, marbofloxacin; ORB, orbifloxacin; PRA, paradofloxacin; PTZ, piperacillin/ tazobactam; SXT, trimethoprim/ sulphamethoxazole; TET, tetracycline; VEC, cefovecin.

Table S4. Antimicrobial resistance patterns of *Escherichia coli* isolated from cats (n = 206) during 2020–2022 in South Korea

Antimicrobials (isolates)	Resistance patterns (isolates)
0 (n = 87)	–
1 (n = 61)	AMP(n=2), LEX(n=58), SXT(n=1)
2 (n = 6)	AMP LEX(n=4), DOX TET(n=1), LEX SXT(n=1)
3 (n = 12)	AMP CHL SXT(n=1), AMP CHL TET(n=1), AMP DOX TET(n=2), AMP LEX CFZ(n=3), AMP LEX ORB(n=1), AMP LEX TET(n=1) LEX CHL GEN(n=1), LEX DOX TET(n=2)
4 (n = 7)	AMP LEX CHL TET(n=1), AMP LEX DOX TET(n=4), AMP LEX GEN SXT(n=1), LEX CHL DOX TET(n=1)
5 (n = 8)	AMP CHL GEN TET SXT(n=1), AMP ENO MAR ORB PRA(n=1), AMP LEX CFZ DOX TET(n=1), AMP LEX CFZ VEC CPD(n=2), AMP LEX CHL DOX TET(n=2), LEX ENO MAR ORB PRA(n=1)
6 (n = 6)	AMC AMP LEX CFZ VEC CPD(n=1), AMP LEX CFZ CHL DOX TET(n=3), AMP LEX CFZ VEC CPD CAZ(n=1), AMP LEX DOX GEN TET SXT(n=1)
7 (n = 6)	AMC AMP LEX CFZ VEC CPD CAZ(n=4), AMC AMP LEX CFZ VEC CPD CHL(n=1), AMP CHL ENO MAR ORB PRA TET(n=1)
8 (n = 2)	AMC AMP LEX CFZ VEC CPD DOX TET(n=1), AMP LEX CFZ VEC CPD GEN TET SXT(n=1)
9 (n = 3)	AMP CFZ CHL ENO MAR ORB PRA TET SXT(n=1), AMP LEX CFZ VEC CPD ENO MAR ORB PRA(n=2)
10 (n = 2)	AMP LEX CFZ VEC CPD CAZ DOX ORB TET SXT(n=1), AMP LEX DOX ENO GEN MAR ORB PRA TET SXT(n=1)
11 (n = 2)	AMP LEX CFZ VEC CPD CAZ ENO GEN MAR ORB PRA (n=1), AMP LEX CFZ VEC CPD DOX ENO MAR ORB PRA TET (n=1)
12 (n = 1)	AMP LEX CFZ VEC CPD CHL DOX ENO MAR ORB PRA TET (n=1)
13 (n = 0)	–
14 (n = 3)	AMC AMP LEX CFZ VEC CPD CAZ CHL ENO MAR PRB PRA TET SXT(n=1), AMP LEX CFZ VEC CPD CAZ DOX ENO GEN MAR ORB PRA TET SXT(n=2)

AMC, amoxicillin; AMP, ampicillin; CAZ, ceftazidime; CFZ, cefazolin; CHL, chloramphenicol; CPD, cefpodoxime, DOX, doxycycline; ENO, enrofloxacin; GEN, gentamicin; LEX, cefalexin; MAR, marbofloxacin; ORB, orbifloxacin; PRA, paradofloxacin; SXT, trimethoprim/ sulphamethoxazole; TET, tetracycline; VEC, cefovecin.
